# Comparison and Optimization of Different Extraction Methods of Bound Phenolics from Jizi439 Black Wheat Bran

**DOI:** 10.3390/foods11101478

**Published:** 2022-05-19

**Authors:** Xi Chen, Kuijie Sun, Kun Zhuang, Wenping Ding

**Affiliations:** 1 Key Laboratory of Bulk Grain and Oil Deep Processing Ministry of Education, Wuhan Polytechnic University, Wuhan 430023, China; xchen@whpu.edu.cn (X.C.); zhuangkunzk@163.com (K.Z.); 2 School of Food Science and Engineering, Wuhan Polytechnic University, Wuhan 430023, China; kuijiesun@163.com

**Keywords:** response surface methodology, ultrasound, microwave, extraction, Jizi439 black wheat, bound phenolics

## Abstract

Diet rich in phenolics would potentially associate with multiple health benefits. Response surface methodology (RSM) was introduced to optimize the process of ultrasound- and microwave-assisted extraction of bound phenolics from the bran of a newly developed black wheat breeding line Jizi439 and then compared with the traditional alkaline method. The optimum conditions were found to be 66 °C, 48 min, and power 240 W for ultrasound-assisted extraction (UAE), and 120 s, power 420 W for microwave-assisted extraction (MAE), respectively. Total bound phenolic contents (TBPCs), determined by Folin-Ciocalteu reagent, were 8466.7 ± 240.9 μg gallic acid equivalents per gram (μg GAE/g) bran for UAE and 8340.7 ± 146.7 μg GAE/g bran for MAE under optimized conditions, which were both significantly higher than that of the traditional method (5688.9 ± 179.6 μg GAE/g) (*p* < 0.05). Antioxidant activities (AAs) were determined by DPPH and ABTS methods. UAE extracts showed the highest DPPH scavenging activity (77.5 ± 0.9%), while MAE extracts showed the highest ABTS scavenging activity (72.1 ± 0.6%). Both were significantly higher than that of the traditional method (69.6 ± 1.1% for DPPH and 65.9 ± 0.5% for ABTS) (*p* < 0.05). Total bound phenolics (TBPs) profiles were further analyzed by HPLC, and results indicated that ferulic acid was dominant, followed by vanillic acid and p-coumaric acid. The contents of each identified individual phenolics were significantly increased by ultrasound and microwave. In conclusion, UAE and MAE were comparable with each other in TBP yields and AAs; however, when taking operation time and energy consumption into consideration, MAE was more efficient than UAE. Our study suggested efficiency extraction methods for further use of bound phenolics as a healthy food ingredient.

## 1. Introduction

Wheat bran, an important cereal industry by-product, is mostly used as fertilizer or discarded, resulting in a great waste of resources [1]. Due to an increased interest in the health benefits of its bioactive phytochemicals, particularly phenolics, the use of bran as a functional food ingredient, is on the rise [2,3,4]. Among different wheat varieties, black wheat was reported to possess the highest level of phenolic content, as well as antioxidant activity, compared with white and purple wheat [5]. Around 80% of the total phenolics in whole grains exist in bound form and are distributed in the bran [6,7,8]. Bound phenolics have shown significantly higher antioxidant capacity than free phenolics in vitro [9,10]. However, studies regarding phenolics are mostly focused on free phenolics rather than their bound form [11,12]. Moreover, the majority of published papers related to bound phenolics in foods mainly include fruits, vegetables, and legumes/seeds [13]. There is a lack of knowledge regarding bound phenolics in grains, such as black wheat bran (BWB). In this respect, a recently developed black wheat Jizi439, which was hybrid from four different breeding lines [14], was selected and investigated in the present study.

To maximize the TBPs yields of Jizi439 BWB and thereby improve their antioxidant activity, response surface methodology (RSM) was introduced and different extraction methods, including the traditional alkaline method, ultrasound-, and microwave- assisted extraction method, were compared in our study. The traditional acid or alkaline method was established in 1982 [15]. Although acid treatment breaks glycosidic bonds of sugars and leaves ester bonds of bound phenolics intact [16], the acid environment may lead to the degradation of flavanols [17]. Compared with acid extraction, alkaline hydrolysis avoids flavanol degradation [18] and reduces their loss [19,20]. However, both methods still have their drawbacks, such as low yield of phenolics and long production cycle. Therefore, a variety of new extraction techniques have been developed, such as ultrasound- [21,22,23] and microwave-assisted extraction [24,25]. These two new green extraction techniques not only greatly shorten the extraction time, but also consume less solvents and energy [26,27]. The acoustic cavities, as well as the mechanical effect created by ultrasound waves, could disrupt the plant cell walls and release the bioactive compounds into solvent [28]. Meanwhile, microwaves possess the ability to heat up the molecules in the plant by ionic conduction and dipole rotation, resulting in the rupture of cell walls, and accelerate the release of phenolic compounds [29]. Extraction of bioactive compounds using ultrasound or microwave has been proven to be an efficient method that reduces the use of toxic chemicals [26,27].

Therefore, the objective of this study was: (1) to optimize UAE and MAE processes using RSM; (2) to compare the TBPC yields, AAs, and the profiles of bound phenolics obtained from UAE, MAE, and traditional alkaline extraction methods under the optimal extraction conditions; and (3) eventually to find an most efficiency way for obtaining more bound phenolics with high antioxidant activity. Taken together, our study fills the gap in optimization of extracting bound phenolics from BWB, provides a fundamental knowledge of bound phenolics profile in the Jizi439 BWB, and suggests an efficient extraction method for further use as a sustainable healthy food ingredient.

## 2. Materials and Methods

### 2.1. Materials and Reagents

The Jizi439 BWB was received from Yueqing Agricultural Science and Technology Co., Ltd. (Handan, China). Tris-HCl buffer was obtained from Solarbio Science and Technology Co. (Beijing, China). DPPH (2,2-diphenyl-1-picrylhydrazyl) and 2,2′-Azinobis (3-ethylbenzothiazoline-6-sulfonate) (ABTS) free radical were acquired from Sigma-Aldrich (St. Louis, MO, USA). Folin-Ciocalteu reagent and NaCO_3_ were purchased from Hushi chemical Co. (Shanghai, China). Standards, including Gallic acid, p-hydroxybenzoic acid, vanillic acid, p-coumaric acid, ferulic acid, syringic acid, caffeic acid, protocatechuic acid, chlorogenic acid, catechin, luteolin, and apigenin, were purchased from Shanghai Yuanye Bio-Technology Co., Ltd. (Shanghai, China).

### 2.2. Preparation of Samples

Milling of Jizi439 BWB used a high-speed multifunctional pulverizer (RHP-100, Jinhua, China) before sieving through a 60-mesh screen. A total of 200 g of bran powder was then transferred to a flask, defatted with hexane at a ratio of 4:1 (*v*/*w*), and then kept on a thermostatic oscillator for 1 h at room temperature. After that, the defatted bran powder was dried in a hood overnight and stored at −20 °C before analysis.

### 2.3. Experimental Design

**Figure 1:**
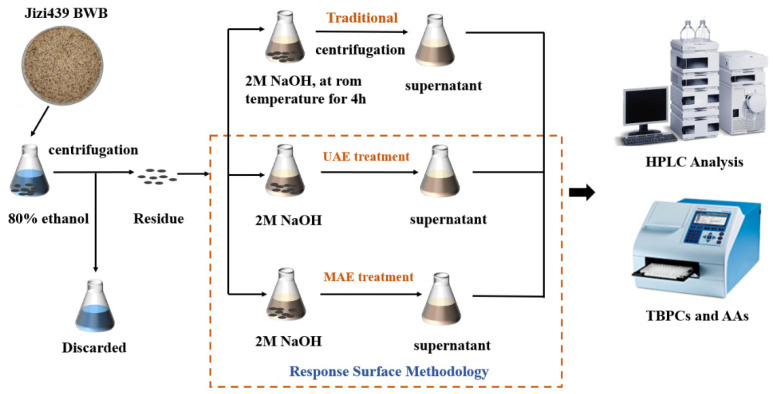
Methodological schematic for the extraction of bound phenolics by the traditional extraction method, UAE, and MAE; UAE, ultrasonic-assisted extraction; MAE, microwave-assisted extraction; TBPCs, total bound phenolic contents; AAs, antioxidant activities.

The scheme of the Jizi439 BWB extraction process, either by traditional, UAE, or MAE methods, was shown in Figure 1. RSM was used to optimize the extraction conditions for UAE and MAE. The defatted bran powder acquired from Section 2.2 was assigned to three groups, including: (1) the traditional alkaline extraction method group, where TBPs were extracted by the traditional alkaline method, without ultrasound or microwave treatment; (2) ultrasound-assisted extraction (UAE), where TBPs were extracted with ultrasound treatment; (3) microwave-assisted extraction (MAE), where TBPs were extracted with microwave treatment. TBPCs and their AAs (in terms of DPPH and ABTS scavenging activity) were compared in each group.

### 2.4. Traditional Alkaline Extraction Method

Bound phenolics were extracted according to Brij Verma et al. [8], with modifications. One gram of defatted Jizi439 BWB was extracted two times with 80% ethanol before centrifugation at 2500× *g* for 10 min. The supernatant was discarded, and the residue was mixed with 40 mL of NaOH (2 M), then left to stand for 4 h at room temperature. After that, the mixture was acidified to pH 2 with 6 M HCl. Bound phenolics were then extracted three times with diethyl ether (DE) and ethyl acetate (EA) (1:1 *v*/*v*) by manually shaking before centrifuging at 2500× *g* for 10 min. DE/EA layers were combined, and the supernatants were pooled and evaporated to less than 5 mL. The concentrated samples were reconstituted to a final volume of 15 mL with methanol, then stored at −20 °C until further use.

### 2.5. Response Surface Design for UAE and MAE

#### 2.5.1. Ultrasound-Assisted Extraction (UAE)

Bound phenolics were extracted with 2 M NaOH and assisted with an ultrasound instrument (THC-2B, Jining Tianhua Ultrasound Electronic Instrument Co., Ltd., Jining, China). Ultrasonic temperature (*X*_1_), ultrasonic time (*X*_2_), and ultrasonic power (*X*_3_) were selected as independent variables. TBPCs measured by the Folin-Ciocalteu method were used as a response value (*Y*). The central composite design (CCD) was carried out for the levels of each independent variable. The ANOVA model was established by the Design-Expert version 8.0.6 software (Minneapolis, MN, USA). The response surface independent variables and levels design are shown in Table 1.

#### 2.5.2. Microwave-Assisted Extraction (MAE)

Bound phenolics were extracted from Jizi439 BWB with 2 M NaOH assisted with a microwave oven (700 W, Galanz Microwave Oven Manufacture Co., Ltd., Foshan, China). Microwave power (*X*_1_) and microwave time (*X*_2_) were selected as independent variables, and TBPCs measured by the Folin-Ciocalteu method were used as a response value (*Y*). The central composite design (CCD) was carried out for the levels of each independent variable. The ANOVA model was established by the Design-Expert 8.0.6 software. The response surface independent variables and levels design are shown in Table 2.

#### 2.5.3. Model Fitting

Experimental data were fitted to a second-order polynomial model. The generalized second-order polynomial model used in the response surface analysis was as follows:

$$Y=\beta_{0}+\sum_{i=1}^{3}\beta_{i}X_{i}+\sum_{i=1}^{3}\beta_{ii}X_{i}^{2}+\sum \sum_{i<j=1}^{3}\beta_{ij}X_{i}X_{j}\;$$

where *Y* represented the measured response (TBPCs); *β*_0_, *β_i_*, *β_ii_*, and *β_ij_* were the regression coefficients for intercept, linear, quadratic, and interaction terms, respectively; *X_i_* and *X_j_* were the coded values of the *i*th and *j*th independent variables. The variable *X_i_X_j_* represented the first order interaction between *X_i_* and *X_j_* for (*i* < *j*).

#### 2.5.4. Verification of Model

Bound phenolics were extracted under optimized conditions of UAE or MAE, and the actual experimental data and the predicted value calculated by equation in Section 2.5.3 were compared for the purpose of determining the validity of the model.

### 2.6. Determination of the Total Bound Phenolic Contents (TBPCs)

TPBCs were determined using Folin-Ciocalteu reagent, as adapted from Chen et al. [30]. Briefly, in each well of the 96-well plate, an aliquot of the 20 µL sample solution or gallic acid standard solution (0–180 µg/mL) was mixed with 100 µL of the Folin-Ciocalteu reagent. The mixture reacted for 5 min at room temperature before the addition of 80 µL 7.5% NaCO_2_ solution and then kept in the dark for 2 h at room temperature. The absorbance was measured at 750 nm using a microplate reader (EnSpire^®^ Multimode Plate reader, PerkinElmer Management Co., Ltd., Shanghai, China). Each well was repeated six times. The TBPCs was calculated and expressed as micrograms of gallic acid equivalent per gram of BWB (µg GAE/g).

### 2.7. Determination of Antioxidation Activity (AA)

DPPH assay: The DPPH scavenging activity of the Jizi439 BWB extracts was determined according to Shimamura et al. [31]. In brief, the DPPH solution was freshly prepared before each use. In total, 20 μL of the sample solution and 80 μL of Tris-HCl buffer (0.1 M) were mixed before 100 μL of DPPH solution was added. The mixture was then allowed to stand for 30 min in the dark at room temperature. Absorbance was read at 515 nm using a microplate reader (EnSpire^®^ Multimode Plate reader, PerkinElmer Management Co., Ltd., Shanghai, China). The radical scavenging activity in terms of the inhibition ratio was calculated using the following equation:

$$\mathrm{Inhibition}\;\mathrm{ratio}\;(\%)=\left(1-\frac{A_{s}-A_{1}}{A_{c}}\right)\times 100$$

where *A_s_* was the absorbance of DPPH solution and Tris-HCl buffer with the sample solution; *A_c_* was the absorbance of DPPH solution and Tris-HCl buffer with methanol; *A*_1_ was the absorbance of Tris-HCl buffer and the sample solution, with methanol for background absorbance.

ABTS assay: This procedure was conducted according to Wu and Maria et al. [32,33], with some modification. The ABTS•^+^ was generated by reacting an ABTS aqueous solution at 7.4 mM with a K_2_S_2_O_8_ solution at 2.6 mM in the dark for 12−16 h and adjusting the Abs_734 nm_ to 0.700 (±0.020) with a pH 7.4 phosphate buffer solution. The sample solution of 10 μL was mixed with 190 μL of ABTS, and the absorbance was measured under 734 nm after avoiding light 20 min at room temperature. The solution was then shaken for 30 s during measurement to fully mix. The radical scavenging activity in terms of the inhibition ratio was calculated using the following equation:

$$\mathrm{Inhibition}\;\mathrm{ratio}\;(\%)=\left(1-\frac{A_{1}-A_{2}}{A_{3}}\right)\times 100$$

where *A*_1_ was the absorbance of the ABTS solution with the sample solution; *A*_2_ was the absorbance of the sample solution with methanol; *A*_3_ was the absorbance of the ABTS solution with methanol.

### 2.8. HPLC Analysis of Bound Phenolic Extracts from Jizi439 BWB

HPLC analysis was carried out according to Kim et al. [18], with slight modifications. The profile of TBPs was analyzed using a Waters e2695 HPLC system equipped with an auto sampler, a Waters XSelect^®^ HSS T3 column (250 × 4.6 mm, 5 µm), and a UV detector (Milford, MA, USA). The mobile phase consisted of acetonitrile (solvent A) and 2% acetic acid solution(solvent B). The elution program was set as follows: 0–30 min, 0–15% A; 30–50 min, 15–50% A; 50–55 min, 50–100% A; 55–60 min, 100–0% A; 60–70 min, 0%A, at a flow rate of 1 mL/min. The injection volume and column temperature were set at 10 µL and 30 °C, respectively. Peaks were read at 280 nm. Retention times and chromatographic peaks were matched with those of the standards.

### 2.9. Statistical Analysis

All measurements were independently conducted in triplicate. Data were expressed as mean ± standard deviations (SD). The adequacy of the RSM model was predicted through the regression (R^2^) and ANOVA analysis. The CCD and the corresponding analysis of the data, including the generated 3D response surfaces and the determination of the optimal set of variables that maximize the yield of bound phenolics, were carried out using the software package Design Expert Version 8.0.6 software (Minneapolis, MN, USA).

## 3. Results and discussion

### 3.1. Modeling Fitting

#### 3.1.1. Modeling of UAE

The data of TBPCs obtained from the 20 experimental runs are shown in Table 3, analyzed by ANOVA and R^2^ interpretation at 95% confidence level (*p* < 0.05) (Table 4) and fitted to a second-order polynomial model (*Y*—TBPCs value, *X*_1_—ultrasonic temperature, *X*_2_—ultrasonic time, *X*_3_—ultrasonic power) with insignificant items being removed:

*Y* = −11,507.48197 + 327.15102*X*_1_ + 177.30934*X*_2_ − 0.38409*X*_1_*X*_3_ − 1.77471*X*_1_^2^ − 1.51980*X*_2_^2^.

The ANOVA in Table 4 declared that the fitted model was significant (F = 29.82, *p* < 0.0001). The insignificance of the lack-of-fit test (*p* = 0.1266 > 0.05) verified the suitability of the selected model. The ANOVA table showed that TBPCs were significantly influenced by ultrasonic temperature (*X*_1_), ultrasonic time (*X*_2_), and the interaction between temperature and ultrasonic power (*X*_1_*X*_3_) (*p* < 0.05, Table 4). The R^2^ value of 0.9641 and adjust R^2^ value of 0.9318 indicated that the observed response values were highly correlated with the predicted values. All the above results revealed the validity of the model for predicting the real correlations between the response and independent variables.

#### 3.1.2. Modeling of MAE

The data of TBPCs obtained from the 13 experimental runs are shown in Table 5, analyzed by ANOVA and R^2^ interpretation at 95% confidence level (*p* < 0.05) (Table 6) and fitted to a second-order polynomial model (*Y*—TBPCs value, *X*_1_—microwave power, *X*_2_—microwave time) with insignificant items being removed:

Y = −2791.25618 + 34.97056*X*_1_ + 55.50481*X*_2_ − 0.035451*X*_1_^2^.

The results of ANOVA for TBPCs are shown in Table 6, which confirm the reliability of the predictive models. The ANOVA table shows that TBPCs were significantly influenced by microwave power (*X*_1_), microwave time (*X*_2_), and the quadratic parameter (*X*_1_^2^) (*p* < 0.05, Table 6). In this experiment, for TBPCs, the R^2^ value was 0.9558, adjust R^2^ value was 0.9242, lack of fit value was insignificant (*p* = 0.0716 > 0.05), and the *p*-value of the model was significant (*p* = 0.0001 < 0.05). All of these indicated that the polynomial prediction model was reliable.

### 3.2. Analysis of Response Surface

#### 3.2.1. Graphical Analysis of UAE

**Figure 2:**
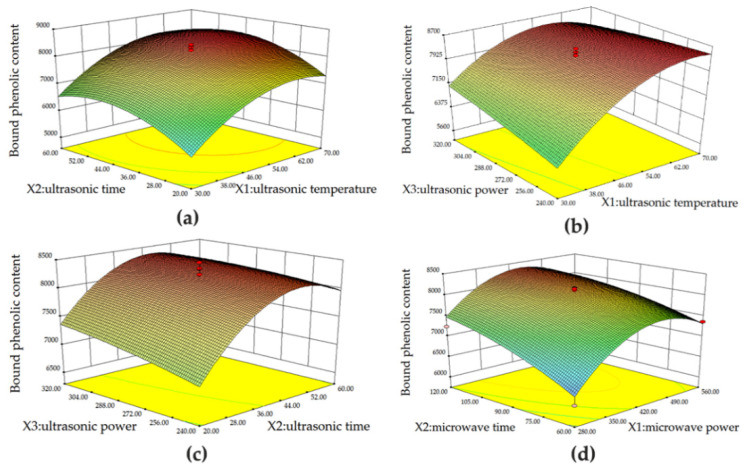
Graphical analysis of the effects of ultrasonic temperature (X_1_, °C) and ultrasonic time (X_2_, min) (**a**); ultrasonic temperature (X_1_, °C) and ultrasonic power (X_3_, W) (**b**); ultrasonic time (X_2_, min) and ultrasonic power (X_3_, W) (**c**); and microwave power (X_1_, W) and microwave time (X_2_, s) (**d**) on TBPCs (μg GAE/g).

The effect of ultrasonic temperature and time on the TBPCs were shown in Figure 2a. The increase in ultrasonic temperature elevated the TBPCs value, which reached peak at around 66 °C, then gradually came to steady. Our results were in agreement with Wang et al. [22], who reported that the total phenolic content was maximized at 65 °C. This was mainly due to the fact that a higher temperature might improve the solubility of phenolic compounds in wheat bran. Similarly, longer extraction time lead to a higher TPBC. It reached the peak at around 52 min and then decreased, which was in line with earlier studies that the highest yields of phenolic compounds are obtained when the ultrasound extraction time is around 55 min [34]. As is shown in Figure 2b,c, increased extraction temperature and time resulted in a remarkable increase in TBPCs, while the ultrasonic power only had a mild influence on the TBPCs (*p* > 0.05, Table 4). These results further confirm that ultrasonic temperature and time significantly affect the response value (TBPC) rather than ultrasonic power.

#### 3.2.2. Graphical Analysis of MAE

The interaction between microwave power and microwave time against the TBPCs value is plotted in Figure 2d. TBPCs increased by extraction time and reached a peak value at 120 s. Meanwhile, the TBPCs value was increased markedly when the microwave power increased from 280 to 420 W. The increasement could be attributed to the exposure of the bran to microwave irradiation, which caused more ruptures in the tissues and cell walls of the bran, leading to an increased release of bound phenolics [35,36]. Additionally, the elevated temperature caused by microwave power increased the mass diffusion rate, which also resulted in more bound phenolics liberated from the bran. However, the long exposure in the presence of high microwave power in turn resulted in a declined extraction rate due to thermal degradation [35,37] that further caused a decrease in TBPCs after 420 W.

### 3.3. Validation and Verification of Predicted Model

#### 3.3.1. Verification of the Model for UAE

The optimal conditions of UAE obtained using the RSM model were as follows: ultrasonic temperature, 65.62 °C; ultrasonic time, 48.28 min; and ultrasonic power, 240 W. However, in practice, it was not applicable to maintain the recommended conditions during processing. Therefore, optimum conditions were targeted as 66 °C, 48 min, and 240 W ultrasonic power. Under the optimal conditions, the model predicted a maximum response of 8605.1 μg GAE/g. Meanwhile, experimental rechecking was performed to compare with the predicted data. A mean value of 8466.7 ± 240.9 μg GAE/g (*n* = 3) was obtained from the real experiments, and the difference between the predicted value and the experimental result was 1.6%, which validated the RSM model.

#### 3.3.2. Verification of the Model for MAE

Based on the prediction of the models, the optimized MAE conditions were microwave power of 420 W and microwave time of 120 s. Under the optimal conditions, the actual TBPC was 8340.7 ± 146.7 μg GAE/g, which was comparable with the predicted value (8362.77 μg GAE/g), and the difference between them was 0.3%, indicating the accuracy of the model.

### 3.4. Comparisons of TBPCs and AAs among Different Extraction Methods

**Figure 3:**
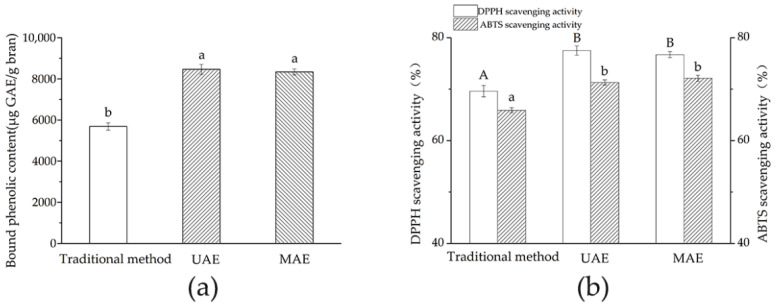
Effect of different treatments on TBPCs (**a**) and antioxidation activity (**b**) of Jizi439 BWB. Values with different lowercase letters indicated statistically significant difference among (**a**) TBPCs or (**b**) ABTS scavenging activities from different treatments (*p* < 0.05). Values with different uppercase letters indicated statistically significant difference among (**b**) DPPH scavenging activities from different treatments (*p* < 0.05).

TBPCs of different extraction methods and their antioxidant activities were compared (Figure 3). According to Figure 3a, although no significant difference was found between UAE and MAE groups, their TPBCs were both significantly higher than that of the traditional method. Similar results were reported when comparing MAE with conventional extraction techniques, regarding extraction of phenolics from blueberry leaves, lemon myrtle, and melastoma sanguineum [38, 39,40]. In addition, TBPCs may vary with different plant sources or extraction methods. For example, Brij Verma et al. [8] reported that TBPCs in 51 wheat cultivars ranged from 2304.9 to 5386.1 µg GAE/g by the traditional alkaline extraction method. Ahmad et al. [41] found that the phenolics from defatted wheat germ obtained by MAE was higher than that of UAE and conventional extraction techniques.

The antioxidant activities of the extracts (in terms of DPPH and ABTS radical scavenging activities) obtained by the three methods were shown in Figure 3b. A higher percentage of DPPH and ABTS scavenging activity is associated with a stronger antioxidant activity. In the present study, remarkable DPPH or ABTS radical scavenging capacity were obtained in all methods. Antioxidant capacities measured by DPPH and ABTS and were not significantly different between UAE and MAE. However, they were both significantly higher than that of the traditional method. Our results confirmed that the DPPH radical scavenging activity of Jizi439 BWB is associated with the content of bound phenolics, which is consistent with previous research [17,42].

Taken together, UAE and MAE rivalled each other in TBP yields and antioxidant activities; however, when taking operation time and energy consumption into consideration, MAE was more efficient than UAE.

### 3.5. HPLC Analysis of Phenolic Profile

**Figure 4:**
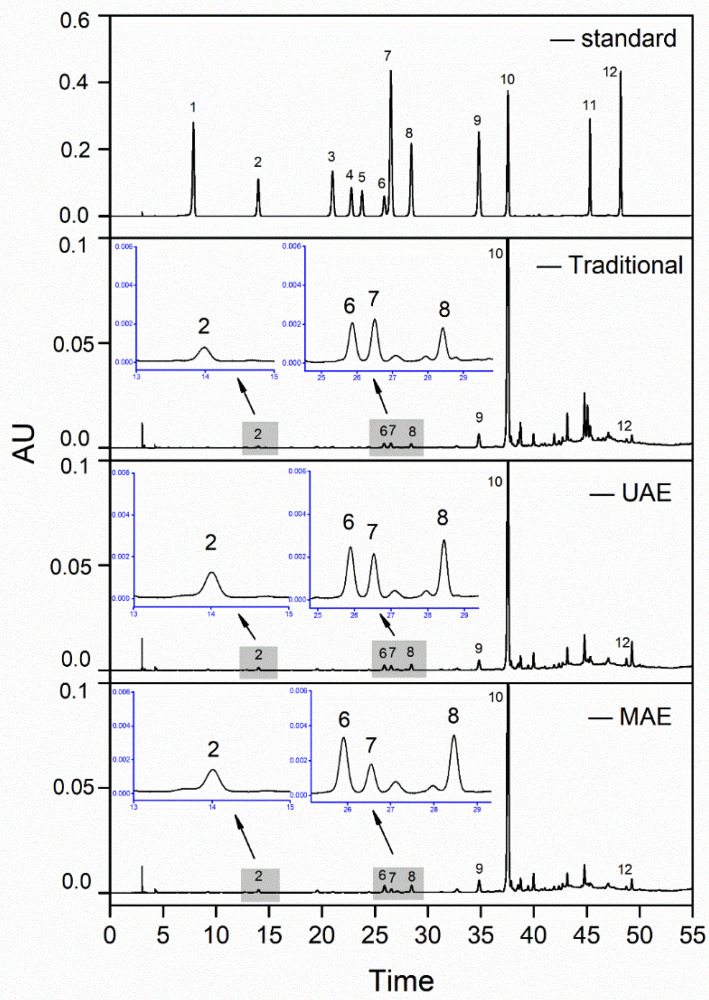
HPLC chromatogram of mixed standards and bound phenolic extracts obtained via different methods. Peak 1: gallic acid; peak 2: protocatechuic acid; peak 3: p-hydroxybenzoic acid; peak 4: catechin; peak 5: Chlorogenic acid; peak 6: vanillic acid; peak 7: caffeic acid; peak 8: syringic acid; peak 9: p-coumaric acid; peak 10: ferulic acid; peak 11: luteolin; peak 12: apigenin.

Qualitative and quantitative analysis of bound phenolics from the extracts obtained by the three methods under optimal conditions were performed using HPLC. Results are shown in Figure 4 and Table 7. The selected 12 standard phenolics have been previously reported in wheat bran [18,30,43], among which six phenolic acids and one flavonoid were detected, including protocatechuic acid, vanillic acid, caffeic acid, syringic acid, p-coumaric acid, ferulic acid, and apigenin. In general, UAE and MAE were more efficient in releasing the phenolic compounds when compared with the traditional method (Table 7). Ferulic acid was predominant in Jizi439 BWB and drastically higher than any other phenolics detected, accounting for 85–91% of the total identified phenolics. Similar phenomena have been mentioned in previous reports [17,44,45]. In addition, our previous study [28] found that ferulic acid was not detectable in its free form in the extract, indicating that ferulic acid existed in bound form in the wheat bran.

According to Table 7, protocatechuic acids and apigenin were highest in UAE extract, meanwhile syringic acid and vanillic acid were highest in MAE extract, and all of them were higher than that of the traditional method. After reviewing published literature, we found that the underlying reasons for the difference in the yield of individual phenolics by UAE and MAE have not been well studied; thus, more investigations should be done in the future. It should be noted that the total amount of identified phenolics was significantly lower than the result obtained by the Folin-Ciocalteu method, because the Folin-Ciocalteu assay does not only measure phenolics, but also reacts with other antioxidants that may exist in the extracts, such as proteins, carbohydrates, and amino acids [46]. In addition, the HPLC only detected the content of several representative phenolics, which may be another reason for the difference in total phenolic content between the Folin-Ciocalteu method and the HPLC.

## 4. Conclusions

The optimal UAE and MAE conditions generated by RSM models were ultrasonic temperature of 66 °C, ultrasonic time of 48 min, ultrasonic power of 240 W for UAE, microwave power of 420 W, and extraction time of 120 s for MAE, respectively. Under optimal conditions, the TBPCs and antioxidant activity of UAE and MAE were comparable with each other, and both were significantly higher than that of the traditional method. However, MAE possessed the economic advantage of a short extraction time with less energy consumption as compared to UAE and the traditional method. Based on our results, MAE is considered an efficient method to obtain large quantities of bound phenolics for further usage of a functional ingredient for food and pharmaceutical products.

## Figures and Tables

**Table 1:** Coded and actual levels of independent variables used in central composite design for ultrasound-assisted extraction.

Factors	Level				
	−1.682	−1	0	1	1.682
*X*_1_	16.36	30	50	70	83.64
*X*_2_	6.36	20	40	60	73.64
*X*_3_	212.73	240	280	320	347.27

*X*_1_, ultrasonic temperature, °C; *X*_2_, ultrasonic time, min; *X*_3_, ultrasonic power, W.

**Table 2:** Coded and actual levels of independent variables used in the central composite design for microwave-assisted extraction.

Factors	Level				
	−1.414	−1	0	1	1.414
*X*_1_	222.01	280	420	560	617.99
*X*_2_	47.57	60	90	120	132.43

*X*_1_, microwave power, W; *X*_2_, microwave time, s.

**Table 3:** The UAE experimental design in coded and uncoded form for the optimization of variables using CCD.

No.	Extraction Conditions (Independent Variables)			Observed Responses (Dependent Variables)
	*X*_1_	*X*_2_	*X*_3_	TBPCs
1	30 (−1)	20 (−1)	240 (−1)	5346.9 ± 66.7
2	70 (1)	20 (−1)	240 (−1)	7564.5 ± 83.8
3	30 (−1)	60 (1)	240 (−1)	6226.5 ± 139.6
4	70 (1)	60 (1)	240 (−1)	8234.5 ± 73.3
5	30 (−1)	20 (−1)	320 (1)	6502.2 ± 104.4
6	70 (1)	20 (−1)	320 (1)	7351.1 ± 94.3
7	30 (−1)	60 (1)	320 (1)	6874.1 ± 154
8	70 (1)	60 (1)	320 (1)	7792.6± 230.5
9	16.36 (−1.682)	40 (0)	280 (0)	4573.3 ± 110
10	83.64 (1.682)	40 (0)	280 (0)	7568.5 ± 178.1
11	50 (0)	6.36 (−1.682)	280 (0)	5329.8 ± 231.6
12	50 (0)	73.64 (1.682)	280 (0)	7388.8 ± 198
13	50 (0)	40 (0)	212.73 (−1.682)	7762.2 ± 33
14	50 (0)	40 (0)	347.27 (1.682)	8123.7 ± 219.9
15	50 (0)	40 (0)	280 (0)	8345 ± 230.4
16	50 (0)	40 (0)	280 (0)	8352.1 ± 125.7
17	50 (0)	40 (0)	280 (0)	8245.7 ± 104.7
18	50 (0)	40 (0)	280 (0)	8453.1±324.8
19	50 (0)	40 (0)	280 (0)	8246.7 ± 199.1
20	50 (0)	40 (0)	280 (0)	7846.6 ± 197.9

*X*_1_, ultrasonic temperature, °C; *X*_2_, ultrasonic time, min; and *X*_3_, ultrasonic power, W; TBPCs, total bound phenolic contents, μg GAE/g.

**Table 4:** ANOVA of the second polynomial model (UAE) for the TBPCs.

Source	Sum of Squares	df	Mean Square	F Value	*p* Value
Model	2.40 × 10^7^	9	2.67 × 10^6^	29.82	<0.0001
*X*_1_	8.91 × 10^6^	1	8.91 × 10^6^	99.70	<0.0001
*X*_2_	2.49 × 10^6^	1	2.49 × 10^6^	27.81	0.0004
*X*_3_	2.26 × 10^5^	1	2.26 × 10^5^	2.53	0.1431
*X*_1_ *X*_2_	2450	1	2450	0.027	0.8718
*X*_1_ *X*_3_	7.55 × 10^5^	1	7.55 × 10^5^	8.45	0.0156
*X*_2_ *X*_3_	67,748.8	1	67,748.8	0.76	0.4043
*X*_1_ *^2^*	7.26 × 10^6^	1	7.26 × 10^6^	81.27	<0.0001
*X*_2_ *^2^*	5.33 × 10^6^	1	5.33 × 10^6^	59.60	<0.0001
*X_3_^2^*	33,223.47	1	33,223.47	0.37	0.5556
Residual	8.94 × 105	10	89,358.65		
Lack of Fit	6.70 × 10^5^	5	1.34 × 10^5^	3.00	0.1266
Pure Error	2.23 × 10^5^	5	44,688.1		
Cor. Total	2.49 × 10^7^	19			
R^2^	0.9641				
Adjusted R^2^	0.9318				

*X*_1_, ultrasonic temperature, °C; *X*_2_, ultrasonic time, min; *X*_3_, ultrasonic power, W; df, degree of freedom.

**Table 5:** The MAE experimental design in coded and uncoded form for the optimization of variables using CCD.

NO.	Extraction Conditions (Independent Variables)		Observed Responses (Dependent Variables)
	*X*_1_	*X*_2_	TBPCs
1	280 (−1)	60 (−1)	6215.9 ± 31.4
2	560 (1)	60 (−1)	7370.4 ± 10.5
3	280 (−1)	120 (1)	7251.9 ± 52.4
4	560 (1)	120 (1)	7851.9 ± 41.9
5	222.01 (−1.414)	90 (0)	6548.2 ± 335.2
6	617.99 (1.414)	90 (0)	7066.7 ± 83.8
7	420 (0)	47.57 (−1.414)	7340.8 ± 73.3
8	420 (0)	132.43 (1.414)	8481.5 ± 408.6
9	420 (0)	90 (0)	8170.4 ± 240.9
10	420 (0)	90 (0)	8126 ± 94.3
11	420 (0)	90 (0)	8163 ± 188.6
12	420 (0)	90 (0)	8155.6 ± 115.2
13	420 (0)	90 (0)	7903.7 ± 52.3

*X*_1_, microwave power, W; *X*_2_, microwave time, s; TBPCs, total bound phenolic contents, μg GAE/g.

**Table 6:** ANOVA of the regression model (MAE) for the TBPCs.

Source	Sum of Squares	df	Mean Square	F Value	*p* Value
Model	5.45 × 10^6^	5	1.09 × 10^6^	30.25	0.0001
*X*_1_	7.74× 10^5^	1	7.74 × 10^5^	21.45	0.0024
*X*_2_	1.23 × 10^6^	1	1.23 × 10^6^	33.97	0.0006
*X*_1_ *X*_2_	76,867.56	1	76,867.56	2.13	0.1877
*X*_1_ *^2^*	3.36 × 10^6^	1	3.36 × 10^6^	93.13	<0.0001
*X*_2_ *^2^*	1.42 × 10^5^	1	1.42 × 10^5^	3.94	0.0874
Residual	2.52 × 10^5^	7	3.61 × 10^4^		
Lack of Fit	2.01 × 10^5^	3	67,092.79	5.25	0.0716
Pure Error	51,156.27	4	12,789.07		
Cor. Total	5.71 × 10^6^	12			
R^2^	0.9558				
Adjusted R^2^	0.9242				

*X*_1_, microwave power, W; *X*_2_, microwave time, s; df, degree of freedom.

**Table 7:** Phenolics composition of extracts from different methods.

Phenolics Standard (μg g^−1^ Bran)	Traditional	UAE	MAE
Ferulic acid	2094.1 ± 9.7 ^a^	2931.7 ± 8.5 ^b^	3084.2 ± 14.5 ^c^
Catechin	ND	ND	ND
Gallic acid	ND	ND	ND
Protocatechuic acid	18.8 ± 1.2 ^a^	60.5 ± 0.6 ^c^	51.1 ± 0.6 ^b^
Syringic acid	20.4 ± 0.4 ^a^	67 ± 1.3 ^b^	71.4 ± 1.6 ^c^
Chlorogenic acid	ND	ND	ND
p-hydroxybenzoic acid	ND	ND	ND
p-coumaric acid	47.1 ± 1.5 ^a^	69.7 ± 2.4 ^b^	85.5 ± 2.6 ^c^
Vanillic acid	91.1 ± 2.4 ^a^	208.4 ± 19.4 ^b^	265.3 ± 2.0 ^c^
Caffeic acid	19.8 ± 1.6 ^a^	39 ± 0.5 ^c^	27.6 ± 0.2 ^b^
Apigenin	8.8 ± 0.9 ^a^	43 ± 0.7 ^c^	11.8 ± 0.1 ^b^
Luteolin	ND	UD	ND
Total	2300.1 ± 1.6 ^a^	3419.5 ± 16.5 ^b^	3596.9 ± 15.3 ^c^

Data were expressed as mean ± standard deviation (*n* = 3). Traditional, TBPs extracted by the traditional alkaline method; UAE, TBPs extracted with ultrasound treatment; MAE, TBPs extracted with microwave treatment. ND, not detected. Values with different lowercase letters within a row differ significantly (*p* < 0.05).

## Data Availability

The data is contained within the article.
